# Quantitative Proteomics of an Amphibian Pathogen, *Batrachochytrium dendrobatidis*, following Exposure to Thyroid Hormone

**DOI:** 10.1371/journal.pone.0123637

**Published:** 2015-06-05

**Authors:** Jose Thekkiniath, Masoud Zabet-Moghaddam, Kameswara Rao Kottapalli, Mithun R. Pasham, Susan San Francisco, Michael San Francisco

**Affiliations:** 1 Department of Internal Medicine, Yale University School of Medicine, New Haven, CT, 06520, United States of America; 2 Center for Biotechnology and Genomics, Texas Tech University, Lubbock, TX, 79402–3132, United States of America; 3 Department of Cell Biology and Pediatrics, Harvard Medical School, Boston, MA, 02115, United States of America, and Program in Cellular and Molecular Medicine, Boston Children’s Hospital, Boston, MA, 02115, United States of America; 4 Department of Biological Sciences, Texas Tech University, Lubbock, TX, 79409–3131, United States of America; University of South Dakota, UNITED STATES

## Abstract

*Batrachochytrium dendrobatidis *(*Bd*), a chytrid fungus, has increasingly been implicated as a major factor in the worldwide decline of amphibian populations. The fungus causes chytridiomycosis in susceptible species leading to massive die-offs of adult amphibians. Although *Bd* infects the keratinized mouthparts of tadpoles and negatively affects foraging behavior, these infections are non-lethal. An important morphogen controlling amphibian metamorphosis is thyroid hormone (T_3_). Tadpoles may be infected with *Bd* and the fungus may be exposed to T_3_ during metamorphosis. We hypothesize that exposure of *Bd* to T_3_ may induce the expression of factors associated with host colonization and pathogenicity. We utilized a proteomics approach to better understand the dynamics of the *Bd*-T_3_ interaction. Using liquid chromatography-mass spectrometry (LC-MS), we generated a data set of a large number of cytoplasmic and membrane proteins following exposure of *Bd* to T_3_. From these data, we identified a total of 263 proteins whose expression was significantly changed following T_3_ exposure. We provide evidence for expression of an array of proteins that may play key roles in both genomic and non-genomic actions of T_3_ in *Bd*. Additionally, our proteomics study shows an increase in several proteins including proteases and a class of uncommon crinkler and crinkler-like effector proteins suggesting their importance in *Bd* pathogenicity as well as those involved in metabolism and energy transfer, protein fate, transport and stress responses. This approach provides insights into the mechanistic basis of the *Bd*-amphibian interaction following T_3_ exposure.

## Introduction

*Batrachochytrium dendrobatidis* (*Bd*), a chytrid fungus, has been implicated in widespread amphibian decline [1–4]. The fungus infects the keratin skin layer of metamorphosed amphibians causing the diseased animals to experience thickening of the epidermal layer and eventually sloughing of the skin [5]. The life cycle of *Bd* consists of substrate-independent motile zoospores and substrate-dependent sporangia [3]. However, little is known about the early events during the fungal-amphibian interaction leading to death of these animals.

The amphibian life cycle primarily consists of tadpole and adult animals, and the transition of tadpole to adult is known as metamorphosis [6]. Thyroid hormone plays an integral role in the metamorphosis of these animals. A strong association of metamorphosis with the rise in circulating plasma concentrations of thyroid hormone has been observed [7]. At the climax stage of metamorphosis, a surge of thyroid hormone occurs, however the level of the hormone is reduced at the end of this stage. Studies have shown that pre-metamorphic tadpoles that are lacking thyroid hormones had the ability to sense exogenous hormone. When pre-metamorphic tadpoles were exposed to thyroid hormone, they were capable of precocious metamorphosis [8], suggesting the importance of the thyroid hormone in the development of amphibians.

Although *Bd* infection negatively affects the feeding behavior of young tadpoles, the infection is not lethal [5], [9]. Various studies have reported massive die-offs of amphibians that have recently undergone metamorphosis due to *Bd* infections [5,10–12]. Additionally, *Bd*-infected amphibians exhibit a weakened immune response to the pathogen. Recent studies demonstrate that *Bd* may cause severe immune suppression in susceptible hosts [13–15]. We recently reported that a *Bd* subtilisin-like serine protease degrades frog anti-microbial peptides [16]. This may lead to increased susceptibility of the host to the fungus.

To better understand the early events during *Bd*-frog interactions, particularly, how *Bd* responds to host-derived thyroid hormone, a study of the proteome was carried out following *in vitro* exposure of *Bd* to thyroid hormone (T_3_). Proteomic analyses have been used to understand pathogenicity in fungi such as *Botrytis cinerea* [17] and *Sclerotinia sclerotiorum* [18]. Additionally, in the human pathogen, *Candida albicans*, a proteomics study demonstrated the importance of proteins involved in hyphal-yeast transitions [19]. Using proteomic and phenotypic profiling of *Bd*, a previous study showed that genotype is associated with virulence [20]. Here we present a global proteomics approach to document protein expression changes in *Bd* exposed to T_3_ and discuss its significance in understanding this fungal-amphibian interaction.

## Methods

### Cultivation of fungus

The VM1 isolate of *Batrachochytrium dendrobatidis* isolated from a diseased Western chorus frog (*Pseudacris triseriata*), was provided by Louise Rollins-Smith (Vanderbilt Univ). *Bd* was maintained on TGhL (1.6% tryptone, 0.2% gelatin hydrolysate, 0.4% lactose, 0.8% agar) plates and all experiments were conducted by inoculation in H-broth (1% tryptone, 0.32% glucose). The fungal culture was routinely maintained at room temperature (21°C) and incubated in the dark.

### Exposure to thyroid hormone

Of the two forms of thyroid hormone, 3, 5, 3'- triiodothyronine (T_3_) and 3, 5, 3', 5'- tetraiodothyronine (thyroxine, T_4_), T_3_ is the biologically more active form in vertebrates [21] and was used in this study. T_3_ was used at the concentration at which it occurs physiologically in tadpoles [22]. A 10 mM stock solution of T_3_ was prepared by dissolving 3, 3′, 5-triiodo-L-thyronine sodium salt (Sigma-Aldrich) in methanol and stored at -20°C. The T_3_ stock was further diluted in water, which was then used for the experiments. The fungal cultures were exposed to a final concentration of 50 nM T_3_ for 3 hours at room temperature. Methanol was used as the solvent control. Three biological replicates of T_3_-treated and control cultures were used for the study.

### Protein extraction

To pinpoint when *Bd* proteins are induced during exposure to T_3_ and at what level they are expressed, a time-course experiment was carried out. Following 7 days of growth in H-broth, *Bd* cells were exposed to a final concentration of 50 nM T_3_ for 1, 3, 6 or 12 hrs. After careful analysis, the 3-hr time point was chosen for this proteomics study. Proteins from *Bd* cells were extracted using a glass bead method. The cells were harvested at 10,000 rpm at 4°C for 10 minutes. The supernatant was discarded and the pellet was resuspended in breaking buffer (50 mM sodium monophosphate (pH 7.4), 1 mM phenylmethylsulfonyl fluoride (PMSF), 1 mM ethylenediaminetetraacetic acid (EDTA) and 5% glycerol). An equal volume of acid-washed glass beads (0.5 mm diameter) was added to the cell suspension. The pellet was then subjected to eight alternate cycles of vortexing and incubation on ice for 30 seconds. The mixture was then centrifuged at 14,000 x g for 10 minutes at 4°C. The resulting supernatant was transferred to a microfuge tube and subjected to 14,000 x g for 10 minutes to further remove any glass beads from the solution. The concentration of total soluble protein was estimated by the Bradford assay [23] and further used for LC-MS study.

### One-dimensional (1-D) gel electrophoresis

Protein samples (100 μg) of 3 biological replicates from T_3_-treated and control were separated on a one-dimensional gel using SDS-polyacrylamide gel electrophoresis (SDS-PAGE). The samples were mixed with protein loading buffer (2% SDS, 1% Tris-HCl, 10% v/v glycerol, 0.01% bromophenol blue, 5% beta-mercapto-ethanol, pH 6.8). The mixture was boiled for 5 minutes, centrifuged briefly and then loaded into a 12% resolving gel (BioRad). Following electrophoresis the gel was stained with 0.1% Coomassie Blue stain for 4 hours followed by destaining (10% methanol, 10% acetic acid, 2% glycerol and 78% water) overnight. The proteins were visualized using Alpha Innotech imaging system (Cell Science).

### In-gel digestion and extraction of peptides

Each sample lane of the gel in which the proteins were separated was cut into 8 equal slices and each slice was kept in a 0.5 ml microfuge tube. In-gel digestion on each slice was carried out as described previously [24]. Briefly, these slices were washed in milli Q water for 5 minutes at 37°C at 600 rpm and then washed twice with acetonitrile (ACN)/100 mM NH_4_HCO_3_ (50/50) for 10 minutes to destain the gels. Subsequently, proteins in the gel pieces were subjected to reduction in 50 μl 10 mM dithiothreitol at 56°C at 600 rpm for one hour. These slices were then washed in water, alkylated in 50 μl of iodoacetamide solution (55 mM in 40 mM NH_4_HCO_3_), and incubated in the dark for 30 minutes. To remove any remaining dye, the gel slices were washed alternately in water and 50% ACN. After completely removing the dye, the gel pieces were incubated for one minute in 100 μl of 100% ACN and air-dried. The samples were digested using 30 μL of sequencing grade trypsin (Promega) solution (12.5 ng/μL in 25 mM NH_4_HCO_3_) and incubated overnight at 37°C. Peptide extraction was performed twice using 50% ACN containing 0.1% formic acid solution. The extracted peptide solutions were pooled and dried using a speed vacuum centrifuge and the peptides resuspended in 20 μl of 0.1% formic acid.

### Nano LC-MS/MS

To analyze the peptides extracted from in-gel digestion, nano-flow liquid chromatography tandem mass spectrometry (nano-LC-MS/MS) was used on an LTQ-XL ion trap mass spectrometer (Thermo, CA, USA) at the Center for Biotechnology and Genomics, Texas Tech University. Chromatographic separation of the peptides was carried out using a Dionex nano-HPLC (Ultimate 3000) with a trapping column (C18, 3 μm, 100 Å, 75 μm × 2 cm) followed by a reverse phase column (C18, 2 μm, 100 Å, 75 μm × 15 cm, nanoViper). Peptides were first injected onto the trapping column, which was equilibrated with 1% ACN, 0.1% formic acid in mass spectrometric grade water. These peptides were trapped for 10 minutes using the loading pump at a flow rate of 5 μl/min. The trapped peptides were then loaded on the reverse-phase analytical column, and bound peptides were eluted using solvents A (2% ACN, 0.1% formic acid in water) and B (98% ACN, 2% water, 0.1% formic acid) at 300 nl/min. The gradient was maintained constant for the first 10 minutes at 4% solvent B followed by a gradual increase up to 30% solvent B in 20 minutes. Solvent B was further increased to 60% in 40 minutes followed by a rapid increase up to 90% over 5 minutes. The eluted peptides were directed into the nanospray ionization source of the LTQ-XL with a capillary voltage of ∼2 kV. The collected spectra were scanned over the mass range of 300–2000 atomic mass units. Data dependent scan settings were defined to choose the 6 most intense ions with dynamic exclusion list size of 100, exclusion duration of 30 seconds, repeat count of 2, and repeat duration of 15 seconds. To generate MS/MS spectra, collision-induced dissociation of the peptide ions at normalized collision energy of 35% was utilized.

### Database search

To identify the proteins using the spectra acquired from the LTQ-XL mass spectrometer, Proteome Discoverer software (version 1.3, Thermo Scientific) was employed. For this purpose, SEQUEST cluster was used as the search engine (Thermo Electron Corp., San Jose CA) against a *Bd* database (www.broadinstitute.org). The following criteria were used by the search engine: precursor ion mass tolerance was set at 2.5 Da, and fragment ion mass tolerance at 0.8 Da. Additional parameters included fully tryptic enzyme specificity, two missed cleavages, and mass range 350–5000 Da and CID as the collision method. For all searches, carbamidomethylation of cysteines and oxidation of methionine were set as dynamic modifications. The false discovery rate (FDR), percentage of false positive identifications among all the tentative peptide identifications, was set at 1% using a decoy databases created from a reversed target database.

### Quantitative proteomic analysis

ProteoIQ software (ProteoIQ 2.70, Premier Biosoft) was used for label-free comparative relative protein quantification using spectral counts. For protein quantification purposes, the following stringent filter criteria were employed: minimum number of spectra = 5, minimum percentage of replicates = 60 (2 out of 3 replicates) and maximum protein false discovery rate (FDR %) = 1. Additionally, probability filters including minimum peptide probability = 0.99 and minimum protein group probability = 0.95 were applied. Using the reversed target database as decoy, the protein FDR was calculated as protein FDR = (number of reverse proteins identified)/ (total protein identifications) x100. To calculate the peptide FDR, the formula, peptide FDR = 2*(number of reverse peptide identifications)/ (total peptide identifications) x100 was used. Protein abundance data were determined using the method described previously [25]. To calculate protein abundance data, normalized spectral abundance factors (NSAF) were employed. In this method, for each protein, k, in sample i, the number of spectral counts (SpC, the total number of MS/MS spectra) identifying the protein was divided by the apparent length of the protein. To calculate the protein length, molecular weight of the protein was divided by the molecular weight of an average amino acid. The NSAFi values for the sample i was determined as SpC_k_/Length_k_ values normalized to the total by dividing by the sum (SpCk/Lengthk). The values in T_3_ normalized spectral count (T_3_ N-SC) Log_2_ relative expression are presented here. To calculate the absolute fold change, the conversion was applied as 2^ (T_3_ N-SC).

### Statistical analysis

To determine the protein expression changes between T_3_ treatment and methanol control, a student t-test was performed. The *t*-test was performed using log transformed NSAF data, and those P-values less than 0.05 were measured to be statistically significant. These protein sets were further subjected to functional annotation.

### Annotation and mapping

To gain evidence on functional annotation of identified *Bd* proteins, the nucleotide sequences of all proteins (Broad Institute) were matched to the NCBI non redundant (NR) protein database and the gene ontology (GO) database by Blast2GO software (Version 1.6) [26]. Using Mercator-Mapman annotation tool, metabolic pathways and other cellular processes in the fungus were mapped (S2 Table and S3 Table). This tool was used to map the differentially expressed proteins into various metabolic pathways [27].

## Results

**Fig 1:**
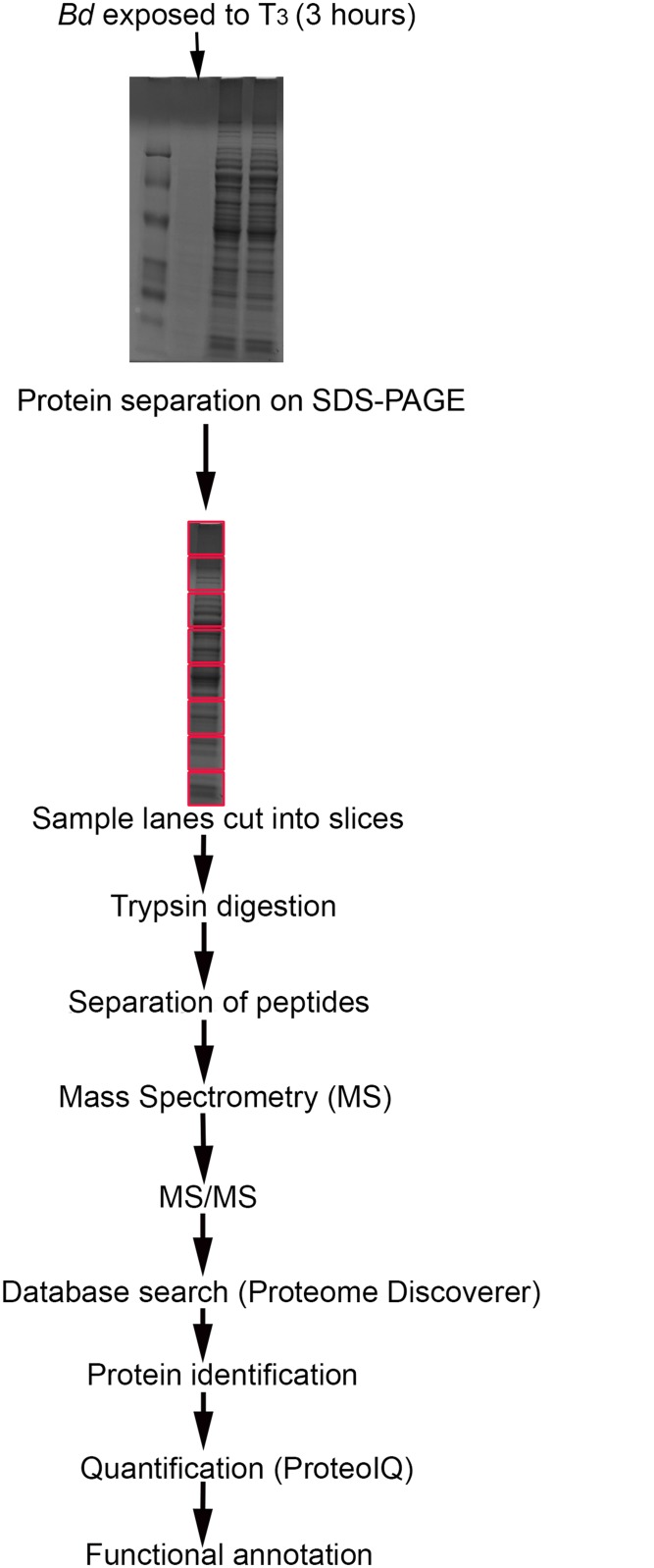
Schematic illustration for the proteomics study to profile total proteins in *Batrachochytrium dendrobatidis* following exposure to T_3_. Red boxes show regions where each sample lane of the gel was cut into slices for peptide extraction.

In an effort to understand the early events during *Bd*-T_3_ interaction, we used a quantitative proteomic approach and assessed the protein expression profiles of *Bd* exposed to T_3_. The strategy followed for protein preparation and profiling using LC-MS approach is shown in Fig 1. Using this approach, we identified and quantitatively analyzed the changes in relative abundance of *Bd* proteins (S1 Table). Among the identified proteins, we found expression differences of 263 proteins that were statistically significant (P value < 0.05). Of these, the expression of 104 proteins was found to have increased by more than 2-fold (Table 1) and 42 proteins were uniquely present (Table 2) in *Bd* cells following exposure to T_3_. We observed a more than 2-fold decrease of 29 proteins (Table 3) while 26 proteins were found to be undetectable (Table 4) in the T_3_-treated samples. Here we list those *Bd* proteins that were, (1) uniquely present or (2) that showed greater than a 2-fold change in abundance (either increase or decrease) following its exposure to T_3_.

**Table 1:** List of proteins that showed more than 2-fold increase in *Bd* exposed to T_3_.

Gene accession number^a^	Name/predicted name of protein	T_3_ (N-SC)Log_2_ relative expression
BDET_08575	prolyl endopeptidase	3.21
BDET_05763	ACTN1 protein	3.1
BDET_04253	hypothetical protein similar to glutamate carboxypeptidase	2.93
BDET_01134	hypothetical protein	2.91
BDET_07031	conserved hypothetical protein	2.9
BDET_02940	rab GDP dissociation inhibitor beta	2.85
BDET_03111	conserved hypothetical protein	2.61
BDET_02571	bifunctional purine biosynthesis protein ADE17	2.46
BDET_04126	bifunctional purine biosynthesis protein ADE17	2.46
BDET_04144	phosphoglucomutase	2.46
BDET_07811	hypothetical protein	2.41
BDET_05116	crinkler family protein	2.4
BDET_05132	crinkler family protein	2.4
BDET_08307	conserved hypothetical protein	2.36
BDET_08030	glucose-6-phosphate isomerase	2.31
BDET_06818	conserved hypothetical protein	2.26
BDET_08009	conserved hypothetical protein	2.24
BDET_04007	hypothetical protein similar to hydrolase	2.2
BDET_01275	electron transfer flavoprotein subunit beta	2.19
BDET_03579	conserved hypothetical protein	2.18
BDET_05121	crinkler family protein	2.16
BDET_01745	aminoacylase-1	2.15
BDET_00627	conserved hypothetical protein	2.14
BDET_07364	conserved hypothetical protein	2.14
BDET_03035	proliferating cell nuclear antigen	2.08
BDET_08526	conserved hypothetical protein	2.08
BDET_05736	triosephosphate isomerase	2.07
BDET_03419	hypothetical protein similar to AhpC/TSA family protein	2.07
BDET_07373	branched-chain-amino-acid aminotransferase, mitochondrial precursor	2.03
BDET_06577	protein disulfide-isomerase erp38 precursor	2.01
BDET_07554	monothiol glutaredoxin-4	2
BDET_02020	long-chain acyl CoA ligase	1.95
BDET_02521	crinkler family missing secretion signal peptide	1.95
BDET_04065	conserved hypothetical protein	1.88
BDET_03580	ribosomal L-30	1.8
BDET_02674	crinkler family missing secretion signal peptide	1.79
BDET_02498	enolase	1.79
BDET_01981	conserved hypothetical protein	1.73
BDET_03190	deoxyuridine 5'-triphosphate nucleotidohydrolase	1.72
BDET_05854	isocitrate dehydrogenase subunit 1, mitochondrial precursor	1.72
BDET_05255	conserved hypothetical protein	1.72
BDET_06670	protein phosphatase PP2A regulatory subunit A	1.71
BDET_02031	hypothetical protein similar to 5'-methylthioadenosine phosphorylase	1.71
BDET_01967	conserved hypothetical protein	1.68
BDET_00885	conserved hypothetical protein	1.67
BDET_00733	4-hydroxyphenylpyruvate dioxygenase	1.64
BDET_06414	multidrug/metal resistance protein, ABC transporter	1.6
BDET_04177	phosphoribosylaminoimidazole carboxylase	1.59
BDET_00864	conserved hypothetical protein	1.59
BDET_01181	hypothetical protein similar to aminopeptidase	1.58
BDET_05762	ATP citrate synthase	1.58
BDET_06464	conserved hypothetical protein	1.54
BDET_04424	hypothetical protein similar to dipeptidyl peptidase III	1.53
BDET_00939	conserved hypothetical protein	1.53
BDET_00944	conserved hypothetical protein	1.53
BDET_06915	conserved hypothetical protein	1.51
BDET_02099	pre-mRNA-processing-splicing factor 8	1.51
BDET_02010	GPI anchor protein	1.5
BDET_06692	NIF 3 like protein	1.5
BDET_06549	tryptophanyl-tRNA synthetase	1.47
BDET_05202	hypothetical protein	1.46
BDET_00751	cell division control protein 3	1.44
BDET_04460	ubiquitin-activating enzyme E1 1	1.42
BDET_08521	peptidyl-prolyl cis-trans isomerase B precursor	1.42
BDET_05202	dihydroorotase	1.4
BDET_06814	cytochrome c peroxidase, mitochondrial precursor	1.39
BDET_02514	predicted protein	1.39
BDET_02516	predicted protein	1.39
BDET_03024	protein phosphatase PP2A regulatory subunit B	1.36
BDET_01885	5-methyltetrahydropteroyltriglutamate-homocysteine methyltransferase	1.35
BDET_04151	glucosamine-fructose-6-phosphate aminotransferase	1.35
BDET_06546	5-methyltetrahydropteroyltriglutamate-homocysteine methyltransferase	1.33
BDET_03560	acetyl-CoA acetyltransferase	1.33
BDET_08315	biofilm development protein YmgB/AriR	1.3
BDET_07061	phosphoglucomutase	1.3
BDET_08261	phosphoribosylglycinamide formyltransferase	1.3
BDET_01150	conserved hypothetical protein	1.29
BDET_01159	conserved hypothetical protein	1.29
BDET_03350	organic hydroperoxide resistance protein	1.29
BDET_02250	conserved hypothetical protein	1.26
BDET_04753	acyl CoA oxidase	1.25
BDET_03317	nucleoside diphosphate kinase 1	1.25
BDET_03057	isocitrate lyase	1.22
BDET_05399	protein phosphatase regulatory subunit SDS22	1.2
BDET_04703	calmodulin	1.2
BDET_06960	conserved hypothetical protein	1.2
BDET_02981	hypothetical protein	1.2
BDET_00091	conserved hypothetical protein	1.18
BDET_07377	ATP synthase F1 gamma	1.1
BDET_05222	heat shock protein 90	1.1
BDET_07409	nucleoside-triphosphatase/ nucleotide binding protein	1.08
BDET_03133	aspartyl-tRNA synthetase	1.08
BDET_01550	conserved hypothetical protein	1.07
BDET_05272	inorganic pyrophosphatase	1.07
BDET_03724	conserved hypothetical protein	1.07
BDET_00078	tubulin alpha-6 chain	1.06

Statistically significant expression at P <0.05.

The values are given as T_3_ normalized spectral count (N-SC) Log_2_ relative expression.

To calculate the absolute fold change, the conversion is applied as 2^ (T_3_ N-SC).

^a^As given according to the www.broadinstitute.org.

**Table 2:** List of uniquely present proteins in *Bd* exposed to T_3_.

^a^Gene accession number	Name/predicted name of protein
BDET_00207	conserved hypothetical protein
BDET_00091	conserved hypothetical protein
BDET_00370	hypothetical protein
BDET_00520	hypothetical protein
BDET_00578	cell division protein kinase 2
BDET_00617	diphosphomevalonate decarboxylase
BDET_01201	succinate dehydrogenase iron-sulfur protein
BDET_01306	hypothetical protein similar to glutathione transferase zeta 1
BDET_01576	cytochrome c1, mitochondrial precursor
BDET_01620	hypothetical protein similar to alcohol dehydrogenase superfamily
BDET_01772	leiomodin-1
BDET_02033	ran-specific gtpase-activating protein 1
BDET_02201	peptidyl-prolyl cis-trans isomerase pin1
BDET_02231	hypothetical protein similar to oxidoreductase
BDET_02645	conserved hypothetical protein
BDET_02736	leukotriene A-4 hydrolase
BDET_02989	predicted protein
BDET_03180	hypothetical protein
BDET_03405	conserved hypothetical protein
BDET_03430	ribonuclease p protein subunit p30
BDET_03541	conserved hypothetical protein
BDET_03674	acyl-binding protein
BDET_03762	valyl-tRNA synthetase
BDET_04022	conserved hypothetical protein
BDET_04238	phosphate induced protein
BDET_04676	xanthine dehydrogenase/oxidase
BDET_05174	novel protein containing Initiation factor 2 subunit family domain
BDET_05329	conserved hypothetical protein
BDET_05479	mt-GrpE
BDET_06040	conserved hypothetical protein
BDET_06411	phosphate induced protein
BDET_06369	predicted protein
BDET_06502	crinkler family protein
BDET_06621	acetoacetyl-CoA synthetase
BDET_06834	alanine aminotransferase 2
BDET_07584	Na/H exchanger
BDET_03192	NADH dehydrogenase ubiquinone alpha
BDET_07703	cytochrome oxidase
BDET_07599	conserved hypothetical protein
BDET_08178	cysteinyl-tRNA synthetase
BDET_08421	10 kDa heat shock protein, mitochondrial
BDET_08720	crinkler family protein

^a^As given according to the www.broadinstitute.org.

**Table 3:** List of proteins that showed 2-fold decrease in *Bd* exposed to T_3_.

^a^Gene accession number	Name/predicted name of protein	T_3_ (N-SC)Log_2_ relative expression
BDET_00978	long-chain acyl-CoA synthetase 7	-2.66
BDET_06102	Rpb (RNA-polymerase)	-2.54
BDET_07602	rRNA 2'-O-methyltransferase fibrillarin	-2.24
BDET_03865	importin beta, transportin	-2.09
BDET_00308	conserved hypothetical protein	-1.97
BDET_06700	dual specificity protein kinase FUZ7	-1.95
BDET_01880	calreticulin	-1.84
BDET_00694	conserved hypothetical protein	-1.83
BDET_03549	CTP synthase	-1.79
BDET_06886	vacuolar sorting protein	-1.59
BDET_08238	shwachman-bodian-diamond syndrome protein	-1.59
BDET_03021	conserved hypothetical protein	-1.50
BDET_07660	26S proteasome subunit p45	-1.39
BDET_05219	pyrD	-1.29
BDET_04706	proline dehydrogenase family protein	-1.29
BDET_03460	60S ribosomal protein L2	-1.24
BDET_04352	glutaminyl-tRNA synthetase	-1.23
BDET_04880	pullulanase	-1.20
BDET_06734	conserved hypothetical protein	-1.20
BDET_08256	G-protein beta	-1.16
BDET_06205	NAD-dependent epimerase/dehydratase family protein	-1.13
BDET_00517	conserved hypothetical protein	-1.16
BDET_07664	conserved hypothetical protein	-1.00
BDET_02151	conserved hypothetical protein	-1.00
BDET_00579	conserved hypothetical protein	-1.00
BDET_01641	conserved hypothetical protein	-1.00
BDET_08055	26S protease regulatory subunit 8	-1.00
BDET_03232	conserved hypothetical protein	-1.00
BDET_00482	polyadenylate-binding protein 1	-1.00

Statistically significant expression at P <0.05.

The values are given as T_3_ normalized spectral count (N-SC) Log_2_ relative expression.

To calculate the absolute fold change, the conversion is applied as 2^ (T_3_ N-SC).

^a^As given according to the www.broadinstitute.org.

**Table 4:** List of uniquely present proteins in *Bd* exposed to methanol control.

^a^Gene accession number	Name/ predicted name of protein
BDET_05257	oligopeptide transporter opt family
BDET_08499	extracellular elastinolytic metalloproteinase
BDET_04809	pre-mRNA splicing factor
BDET_04728	mrna turnover protein 4 homolog
BDET_03527	hypothetical protein
BDET_02501	hypothetical protein
BDET_06402	hypothetical protein
BDET_02113	conserved hypothetical protein
BDET_04582	multiple coagulation factor deficiency isoform
BDET_00183	TatD Dnase family Scn1
BDET_00782	retinoid dehydrogenase
BDET_01194	zuotin
BDET_01995	RNA polymerase
BDET_02205	cyclophilin
BDET_02773	deoxyhypusine hydroxylase
BDET_03497	transporter SEC 24
BDET_03858	expressed protein
BDET_05019	chromosome segregation protein SudA
BDET_05542	septin
BDET_05655	ARM repeat containing protein
BDET_06581	SCF ubiquitin ligase complex subunit CulA
BDET_06582	cullin-1
BDET_06665	transporter SEC 24
BDET_07485	actin-binding protein
BDET_08328	U2 small nuclear ribonucleoprotein A
BDET_01389	conserved hypothetical protein

^a^As given according to the www.broadinstitute.org.

To gain an integrated perspective of the *Bd* biological processes influenced following exposure to T_3_, the complete dataset of identified proteins was classified into Mapman functional categories. Mapman-Mercator analysis has been widely used in analyzing gene expression in higher plants [21] and green algae, *Chlamydomonas reinhardtii* [28]. Mapman pathway analysis in our study showed that *Bd* proteins that were identified are involved in metabolism and energy acquisition, cytoskeleton signaling, and ubiquitin and autophagy-dependent degradation. Such changes in protein expression levels belonging to diverse functional groups point to a broad fungal response when exposed to T_3_.

## Discussion

Our experimental data support the hypothesis that exposure of *Bd* to T_3_ results in protein expression changes associated with various cellular roles in the fungus. This study shows the expression of a large number of proteins in *Bd*, which have been described in both genomic and non-genomic actions of T_3_ in vertebrates. Additionally, this study sheds light on the possible mechanism of how T_3_ may act in *Bd*.

### Genomic action of T_3_ in *Bd*

In vertebrates, in addition to nuclear receptor regulators that control transcriptional activity in a hormone dependent manner, the action of thyroid hormone receptor can be controlled by other proteins [29]. Our study provides evidence for certain cellular proteins in *Bd* that may control the transcriptional activity of thyroid hormone receptor (TR). These TR-interacting proteins primarily consist of transcription modulators and cytoskeletal element regulators such as cyclin dependent kinases, 26 S proteasome subunit p45, ubiquitin-proteasome pathway components including SCF ubiquitin ligase complex subunit culA and cullin, cytoskeletal elements such as actin binding protein, tubulin and alpha-actinin. Additionally, a TR interacting protein- 13 (TRIP-13) (BDET_00690) has been identified in *Bd*. However, expression of this protein did not change following exposure to T_3_.

### Non-genomic action of T_3_ in *Bd*

Membrane receptors play an important role in non-genomic actions of T_3_ [29]. These receptors could be proteins such as integrin or the G-protein coupled receptor (GPCR). Additionally, it has been shown that rapid response to T_3_ is moderated by the mitogen activated protein kinase (MAPK) signaling pathway [30]. Mitogen-activated protein kinases (MAPKs) belong to a family of serine-threonine protein kinases. These kinases are known to play important roles in the signal transduction of a large number of external stimuli and in development and differentiation processes [31]. Like other eukaryotic cells, fungi including yeast and human pathogens such as *Candida albicans* respond to several extracellular stimuli using highly conserved MAPK signaling cascades. Although not statistically significant, we observed a decrease in abundance of the protein Fuz7, a homolog of the yeast protein Ste7 which is found to be involved in the pheromone response pathway in the fungus [32]. In the case of the plant pathogen, *Ustilago maydis*, Fuz7 codes for a MEK/MAPKK homolog. Additionally, this protein has been implicated in a pathway that responds to plant signals [33]. The Fuz7 (BDET_06700) protein is known to be a key protein in the MAPK signaling pathway, which is mediated by G-proteins. It has been shown that GPCRs are involved in this signaling pathway, which results in a corresponding decrease in the receptor and subsequent hormone response. Thus reduced abundance of the Fuz7 protein in this study may be due to desensitization of a GPCR.

### Action of T_3_ on plasma-membrane transport function

#### Na^+^/H^+^ transporter

The sodium-proton exchanger protein, which is known to play an important role in non-genomic action of T_3_ in vertebrates, was among uniquely present proteins in *Bd* exposed to T_3_. A previous study showed that a sodium-proton antiporter in humans was regulated by T_3_ [34]. Sodium-proton exchangers pump Na^+^ ions either out of cells or into cells in exchange for H^+^ [35]. A recent study in two pathogenic species of *Candida* elucidated the role of a membrane Na^+^/H^+^ exchanger in salt tolerance [36]. Since *Bd* lives in fresh water environments, the role of Na^+^/H^+^ exchanger in this fungus is not very clear. However, we have observed that a subtilisin-like protease (SSP), one of the possible pathogenicity factors in *Bd*, requires sodium ions for its optimal activity [12]. Taken together, our observation suggests that a Na^+^/H^+^ transporter (BDET_07584) may be important for *Bd* SSP function possibly during pathogenicity.

#### Ca2^+^ATPase, Calmodulin and Calreticulin

In eukaryotes, Ca^2+^ pumps and transporters have been shown to be important in maintaining the resting cytosolic free Ca^2+^ concentration [Ca^2+^] at very low levels. It has been shown that certain hormones and environmental signals cause a surge in Ca^2+^ concentration which further triggers several downstream signaling proteins such as protein kinase C (PKC) and Ca^2+^ /calmodulin (CAM)- binding kinases [37], [38]. Several studies have demonstrated the role of Ca^2+^ **-** modulated signal cascades in biological processes including, circadian rhythms, differentiation, cell cycle and stress responses in eukaryotic cells [39–42].

Our study showed changes in the abundance level of diverse calcium signaling proteins including Ca^2+^ATPase (BDET_ 06015), calmodulin (BDET_04703) and calreticulin (BDET_01880). We detected a 2-fold increase in abundance of Ca^2+^ATPase, a calcium pump- associated enzyme (Table 1). In eukaryotes, this protein is involved in maintaining intracellular calcium concentration at extremely low levels [43] and the activity of Ca^2+^ ATPase is moderated by thyroid hormone [44]. We also detected a 2-fold increase in calmodulin, a cytoplasmic intracellular Ca^2+^ binding protein, which is involved in the modulation of plasma membrane Ca^2+^ ATPase activity. Calmodulin is also important for the ability of thyroid hormone to enhance the activity of this ATPase [45].

As a Ca^2+^ receptor, calmodulin modulates several intracellular proteins in diverse signaling pathways [46]. Calmodulin has been reported in zoospores of the aquatic chytrid, *Blastocladiella emersonii* [47]. The Ca^2+^-calmodulin complex has been shown to play a key role during growth and sporulation in this fungus [48]. The high abundance of calmodulin following T_3_ treatment in this work suggests its role in maintaining low Ca^+^ levels in *Bd*. Additionally, in fungi; calmodulin has been implicated in stress responses, virulence, and morphogenesis. For instance, in fungal plant pathogens *Magnaporthe grisea* and *Colletotrichium trifolli*, calmodulin has been shown to be essential for the growth of specialized infection structures known as appressoria [49], [50]. Thus the increased abundance of calmodulin following T_3_ exposure supports a role of this hormone in pathogenicity of the fungus.

Calreticulin is an essential Ca^2+^ binding protein in the endoplasmic reticulum [51] and is involved in two major functions in the ER lumen including chaperoning and regulation of Ca^2+^ homoeostasis [52], [53]. Additionally, this protein has been involved in several cellular processes such as cell adhesion, migration and signal transduction [54], [55]. Our observation of a decrease in abundance of calreticulin in response to T_3_ implies that the hormone may cause a significantly reduced Ca^2+^ storage capacity in the ER in the fungus. Our observation also suggests a role for Ca^2+^ in physiological changes in *Bd* including reduced cell motility which may trigger chitin synthase actively favoring the transition from a wall less, motile zoospore to a walled sporangium.

### Action of T_3_ on mitochondria

In the current study, we detected a higher abundance of enzymes involved in mitochondrial oxidative phosphorylation in the T_3_ treatment. For example, proteins that are implicated in oxidative phosphorylation including cytochrome c oxidase (BDET_07703) and NADH dehydrogenase subunit (BDET_03192) are uniquely present following fungal exposure to T_3_. We also observed an increase of 2-fold in the F1-ATPase subunit (BDET__07377) in *Bd* exposed to T_3_. This observation is consistent with that of mammalian systems where T_3_ stimulates mitochondrial respiration resulting in increased ATP production.

### Fatty acid metabolism

Among the proteins that showed a dramatic increase in expression (3.9-fold change) was the long chain acyl CoA ligase (BDET_02020). We also found an increased (2.4-fold change) abundance of acyl CoA oxidase (BDET_04753). The role of fatty acid β-oxidation in fungal pathogenesis is highly suggested by the abundance level of lipid metabolism-associated genes when it infects its host. Successful fungal pathogens, such as *C*. *albicans* utilize proteins for respiratory catabolism such as long chain acyl CoA ligase and acyl CoA oxidase for efficient nutrient acquisition and energy production *in vivo* [56]. Abundance of these enzymes in *Bd* exposed to T_3_ suggests that the hormone treatment may influence fungal acquisition of nutrients or use fatty acids as an energy source.

### Central carbohydrate metabolic processes

Exposure to T_3_ also stimulated expression of proteins involved in carbohydrate metabolism. A protein similar to phosphoglucomutase (BDET_07061) showed a 2.5-fold increase compared to the control. This protein plays a role in the reversible interconversion of glucose-1-phosphate to glucose-6-phosphate. In *Aspergillus nidulans*, phosphoglucomutase has been implicated in asexual development of the fungus [57]. Our observation of an increased expression of phosphoglucomutase suggests that in response to T_3_, this protein may aid the fungus in its developmental processes such as the transition from zoospores to walled reproductive thalli.

### Pathogenicity-associated proteins

Exposure to T_3_ also showed an increase of seven proteins (BDET_06502, BDET_08720, BDET_05121, BDET_05116, BDET_05132, BDET_02674, and BDET_02521) that are highly similar to that of the crinkler family proteins or crinkler-like effectors (CRN) when compared to the control. Microbial effectors are implicated in the destruction of host defenses and thus are capable of changing the host cell metabolism [58]. These proteins were assumed to be present only in Oomycetes, a significant group of pathogens in fish and plants. In general, CRN effectors consist of a signal peptide, a translocator domain that helps the entry of CRN proteins into host cells, and a C-terminal domain that is found to be involved in host protein interaction. These effectors are denoted as crinkler proteins due to their involvement in leaf crinkling and cell death. Interestingly, *Bd* causes similar effects on amphibian skin [59]. The possibility that *Bd* might have acquired these genes from Oomycetes through horizontal gene transfer has been discussed [60]. Our observation suggests that crinkler proteins may play a key role in *Bd* virulence and may be regulated by T_3_. A recent genome-wide study in *Bd* showed an increased expression of *Bd* crinkler and CRN genes in the frog skin. Interestingly, this study also demonstrated a dramatic expression of CRN genes in the *Bd* zoospore as compared to reproductive thalli [61]. Our observation in the current study suggests a comprehensive analysis of CRN genes in *Bd*.

In addition to crinkler proteins, we identified proteins that may be implicated in proteolysis and thus fungal pathogenesis. For example, prolyl endopeptidase (peptidase S9) (BDET_08575) showed a 9.3-fold increase, aminopeptidase P (peptidase M24) (BDET_01181) had a 3-fold increase, and the dipeptidyl peptidase III (peptidase M49) (BDET_04424) had a 2.9-fold increase. Additionally, leukotriene A-4 hydrolase (M1) (BDET_02736), was uniquely present in *Bd* exposed to T_3_. Interestingly, these proteases were also identified in a recent study wherein anuran skin was exposed to supernatant of *Bd* zoospores which caused disruption of intercellular junctions in frog skin [62]. Like other successful pathogens, *Bd* may use proteases to modulate immune responses in amphibians. Genomic analysis of *Bd* has indicated an intense expansion of the fungalysin metallopeptidase and serine peptidase gene families in the fungus [63]. Serine proteases are important virulence factors in parasites and pathogenic microbes [64]. *Bd*-infected *Silurana tropicalis* showed an increase in expression of serine proteases [15] and a recent *in vitro* study showed a *Bd* subtilisin-like serine protease impairs frog antimicrobial peptides [16]. These observations suggest that these proteases are important in impairing vertebrate innate immunity. In this study, we identified a dramatic increase in both metalloprotease and serine peptidase proteins. Thus our study provides further evidence for proteins that may inhibit innate immune responses in the susceptible host species [13], [15], [65]. Previous studies with *Bd*-infected frogs showed a clear down-regulation of adaptive immunity in the animals [13], [15]. A recent study demonstrated *Bd* is capable of impairing host lymphocyte responses and inducing apoptosis [14]. In another study Ellison et al showed that *Bd*-infected frogs had lower expression of many genes involved in adaptive immunity including B-cell related genes, T-cell markers and many T-cell receptor components [66].

Mechanisms by which fungi cause immunosuppression involve the manipulation of host-immune receptors [67] and the discharge of toxins [68]. *Bd* may release toxic components [69] that impede immune responses in *vitro* and these components may be produced from the *Bd* cell wall [14]. In this study, we used whole-cell extracts and thus the proteins identified include mixed life-stages of *Bd* consisting of both cell walled sporangia and wall-less zoospores. A further proteomics study that separates developmental stages of *Bd* (zoospores and sporangia) may provide clearer evidence for gene expression changes associated with immunosuppression caused by *Bd*.

### Heat shock proteins (Hsps)

In the current study, we observed a significant up-regulation of *Bd* proteins that are implicated during survival of stress conditions. These proteins include heat shock proteins Hsp10 (BDET_08421) Hsp 90 (BDET_05222) and Hsp101 (BDET_04470). Hsps are cellular chaperones that play a key role in protein folding homeostasis, revival and degradation of impaired proteins [70], and in thermo tolerance [71] [72]. Hsp90 is a vital and remarkably conserved chaperone in all eukaryotes and controls the role and stability of a variety of proteins including nuclear steroid receptors and protein kinases [73]. A recent study has shown that Hsp90 plays a key role in regulating morphogenetic switch from yeast to hypha and is temperature dependent [74]. In the case of aquatic chytrid fungus, *B*. *emersonii*, the expression of genes encoding cytoplasmic and endoplasmic reticulum Hsp90 proteins (Hsp90A and Hsp90 B) has been documented in response to thermal stress [75]. In addition to response to heat shock at 38°C, the levels of Hsp90A have been increased at physiological temperature (27°C) both during fungal germination and sporulation. The increase in expression of Hsp90 in our study suggests that Hsp90 might be involved in the morphological switch from zoospore to thallus. A recent study reported the up-regulation of several *Bd* Hsps including Hsp90 in frogs infected with *Bd* implying a role of Hsps under stress conditions [11].

Hsp10, a fungal equivalent of *E*. *coli* GroES has been known to function in association with Hsp60 to form a chaperonin that favors mitochondrial folding [76]. Additionally, Hsp10 has been shown to have a role in defending cells from various stresses due to infection and inflammation [77],[78]. In pathogenic fungi such as *C*. *albicans*, the role of Hsp10 remains unknown. The significance of the increase of Hsp10 in our study is not clear; perhaps this protein, like in other eukaryotes, might be involved in mitochondrial protein folding. Hsp101, a member of Hsp100/ClpB family of chaperones is vital for resistance to high temperature stress. The cytosolic Hsp101 in yeast and plants contributes to thermo tolerance [79–81]. It is important to note that Clp proteins are essential to cells that are not only exposed to heat stress, but also other forms of environmental stresses. It has been shown that Hsp101 in yeast, in addition to heat stress confers resistance to chemicals including ethanol and arsenite [82]. The increased abundance of Hsp101 in our study suggests that exposure to T_3_ may create chemical stress condition and that Hsp101 may allow *Bd* to tolerate the host immune response.

### Mapman-Mercator pathway analysis

Mapman analysis revealed a high abundance of enzymes that are involved in amino acid biosynthesis, suggesting that when *Bd* is exposed to T_3_, the fungal cells may require increased protein synthesis for thallus formation. The transition from zoospore to thallus in *Bd* is important for fungal colonization of the host. However, this transition is an energy-expensive process, which requires a large supply of resources for repeated mitotic cell divisions and cell wall synthesis. Amino acids could conceivably be used as an energy source. During the infection process, *Bd* must obtain nutrients from its hosts and the fungus may primarily depend on amino acids generated by proteases. Recent studies have identified and characterized *Bd* proteases that may be involved during infection [16], [83]. In addition to serving as a source of carbon and protein building blocks, amino acids play other roles in fungi. For example, in the case of *Allomyces*, certain amino acids play an essential role in making sugars including fructose and mannose and thus may serve as sources for both carbon and nitrogen [84]. In mammalian systems, iodothyronines such as T_3_ are considered as a special class of amino acids from two tyrosine residues and that amino acid transporters play a key role in thyroid hormone uptake into several tissues [85], [86]. Our results indicate that increased amino acid synthesis in the fungus may further aid in thyroid hormone transport.

Analysis of central metabolic pathways showed a variation in protein abundance changes for those proteins involved in the tricarboxylic acid (TCA) cycle. For example, succinate dehydrogenase (SDH) was found in relative abundance, while malate dehydrogenase (MDH) was less abundant in *Bd* following its exposure to T_3_. The enzymes implicated in the glycolytic pathway were found to be up-regulated. It was also observed that the enzymes involved in the glyoxylate cycle including malate synthase (MS) and isocitrate lyase (ICL) were up-regulated in *Bd* following its exposure to T_3_. The glyoxylate cycle is involved in lipid metabolism and in fungi is a peroxisome-associated process. In *Bd*, as zoospores are released from the zoosporangium, they contain several lipid globules that are partly surrounded by the microbody, a key characteristic of *Bd* [3]. The lipids present in the zoospores might be broken down using the beta-oxidation pathway present on the peroxisome. These degradation products might be further processed through the glyoxylate cycle to maintain growth of new sporangia. A similar observation was also made in a nematode-trapping fungus, *Arthrobotrys oligospora* [87]. Using proteomics and genomics approaches, Yang and coworkers demonstrated the up-regulation of these enzymes in *A*. *oligospora* in response to nematode extract [87]. A previous study has demonstrated that glyoxylate cycle is essential for fungal virulence [88]. Additionally, our current study is consistent with that of *C*. *albicans* in that while the fungus infects macrophages, these key enzymes were found to be up- regulated [89].

In summary, the proteomics data described in our study help to understand cellular responses of *Bd* following its exposure to a host-derived morphogen. This response relies on the expression of a particular group of proteins or genes allowing the fungus to adapt to its environment. Our results implicate proteins involved in metabolism and energy, protein fate, transport, stress responses and pathogenesis in *Bd* that respond to exposure to T_3_. These observations provide a basis for further experimental exploration.

## Supporting Information

S1 Table All identified proteins in *Bd* following exposure to T_3_. (XLS)

S2 Table MapMan annotation of proteins identified in *Bd* following exposure to T_3_. (XLS)

S3 Table Classification of all identified proteins in *Bd* following exposure to T_3_ according to the Mapman-Mercator categories. The identified proteins were classified according to the previously defined Mapman functional categories by using the online Mapman-Mercator annotation tool.
(XLSX)
